# From accelerometer data to activity patterns in chronic pain: methodical reasoning is key

**DOI:** 10.3389/fspor.2026.1712235

**Published:** 2026-02-23

**Authors:** Annet Doomen, Ivan Huijnen, Harriët Wittink, Tale Evenhuis, Martine Verwoerd, Rob Smeets

**Affiliations:** 1 Department of Healthy and Sustainable Living, HU University of Applied Sciences Utrecht, Utrecht, Netherlands; 2 Research School CAPHRI, Department of Rehabilitation Medicine, Maastricht University, Maastricht, Netherlands; 3 Adelante Centre of Expertise in Rehabilitation and Audiology, Hoensbroek, Netherlands; 4 Research Centre on Appropriate Rehabilitation Care, Zuyd University of Applied Sciences, Heerlen, Netherlands; 5 CIR Clinics in Rehabilitation, Eindhoven, Netherlands; 6 Pain in Motion International Research Group (PiM), Brussels, Belgium

**Keywords:** accelerometry, activity pattern, chronic pain, data processing, inertial measurement unit, physical activity, wearable sensors

## Abstract

Activity pattern (AP) concepts are widely used in interdisciplinary chronic pain (CP) care. It is assumed that AP can be objectively measured with accelerometry, but previous results show inconsistencies which may be attributed to methodical choices. This scoping review aims to provide insight in the process of methodical reasoning for measuring AP concepts within CP and the implications for the validity of the methods presented. This process comprises (1) selection of the AP-related concept, (2) its definition or specification (conceptualization), (3) its operationalization with variables and indicators, and (4) measurement properties and data processing. Five databases were searched for full-text publications from inception to November 2024 and publications were included when AP were measured with tri-axial accelerometers for at least five consecutive days in adults with primary musculoskeletal CP. The searches yielded 11,648 papers. Fifteen papers were included and summarized in three tables: 1. Study characteristics, 2. Concepts, definitions and operationalization, 3. Measurement properties and data processing. Concepts were diverse and could be divided into behavioral concepts and concepts derived from physics. Methods were heterogeneous, reporting of methods was incomplete and validity was doubtful in multiple cases. This review provides recommendations for future research steps that could facilitate the comparison of upcoming studies and their application in daily practice. As a first step in securing comparability and usability of future research, consensus is needed on clinically relevant and valid AP concepts and their definitions, followed by consensus on operationalization, data processing, and measurement methods.

**Systematic Review Registration:**

10.17605/OSF.IO/A8U6J.

## 1 Introduction

Chronic pain (CP), defined as pain persisting or recurring for at least three months (1), significantly impacts various aspects of daily life, including physical, mental, social, economic (2, 3) and spiritual (4) domains. Individuals with CP often experience negative emotions, fatigue, depression, deconditioning, sleep dysfunctions and reduced self-efficacy (5). These challenges can lead to limitations in expressing the self, work, leisure and family life and to social isolation (2, 3). For healthcare providers, CP presents a complex challenge due to the interplay of biological, social, and psychological factors. Often, there is no definitive intervention to resolve CP, leading healthcare interventions to focus on self-management and mitigating the negative consequences, such as limitations in activities, participation, and quality of life (6, 7).

It is hypothesized that CP interferes with activity patterns (AP) within daily living (8–13). AP have been defined as the temporal structure of physical activity and sedentary behavior accumulated over a specified period during waking hours (14). Therefore, AP capture the succession of activity and rest, rather than cumulative measures like total activity per day. It is theorized that AP in individuals with CP differ from those in healthy populations, are influenced by coping styles and are associated with various health-related outcomes (9, 15–22). Several theoretical models have been proposed to characterize AP in CP, including the avoidance-endurance model (13).

Traditionally, AP have been assessed using self-report questionnaires. More recently, objective measurement using accelerometry, which is the measurement of acceleration forces with a small body-worn device to detect activity, inactivity and activity intensity, has gained attention (23–25). However, consensus on optimal assessment methods remains lacking. Comparisons of questionnaires with accelerometer outcomes have yielded no or inconsistent associations (26–28). This evokes uncertainty about the validity of questionnaire outcomes for measuring AP on the one hand, and the effects of methodical choices in accelerometry on the other hand (26, 27, 29–31). Self-report instruments are inherently subjective, relying, amongst others, on retrospective recall, personal perceptions and emotional state, which limits their ability in capturing actual behavior. The validity of accelerometry is influenced by incidental user-related factors, such as variations in the wear angle of the sensor.

More importantly, validity of accelerometry is structurally affected by methodical choices, including variables in data processing, and outcome variable selection. For example, very short epoch lengths may introduce unnecessary noise, whereas overly long epoch lengths may obscure temporal detail. Moreover, accelerometer studies often rely on coarse metrics such as total time per day (32–34) which fail to capture the nuanced temporal structure of activity and rest.

To interpret findings from previous studies and relate them to methodical choices, detailed insight into these methods is needed. AP are constructs—abstract representations that require clear definitions and operationalization for empirical investigation. This process involves thoroughly defining the concept followed by its operationalization with measurable variable and outcome measures.

Although recent reviews have explored behavior-related activity parameters from accelerometry (35, 36) and cumulative activity in CP populations (37), no review has specifically focused on AP parameters relevant to CP. Therefore, this scoping review aims to provide an overview of methods for extracting AP parameters relevant to CP care from accelerometer time series. The primary aim is to elucidate the process of methodical reasoning for extracting AP from accelerometer data in patients with CP. This will inform researchers on the usability and availability of methods to investigate AP in patients with CP, and on the underlying concepts and definitions. The secondary aim is to interpret and compare clinically relevant findings in light of methods employed.

The review is structured around the methodical reasoning process, encompassing: (1) selection of the AP-related concept, (2) its definition or specification (conceptualization), (3) the definition of variables and indicators that can be observed and measured (operationalization), and (4) the measurement properties and the methods for extracting the indicators from raw accelerometer data. The overview of conceptualization, operationalization and measurement properties enables qualitative assessment of content validity of the methods included. This scoping review (38) includes studies employing triaxial accelerometers to assess AP in individuals with chronic primary musculoskeletal pain. This condition is defined as CP in the muscles, bones, joints, or tendons that is characterized by significant emotional distress or functional disability, that cannot be accounted for by another condition (39, 40).

## 2 Materials and methods

The research protocol has been published in the Open Science Framework (OSF; OSF | Accelerometry in chronic pain). This scoping review is conducted and reported according to the Preferred Reporting Items for Systematic reviews and Meta-Analyses extension for Scoping Reviews PRISMA-ScR guidelines (41). A scoping review is the most appropriate method to fit our aim, as it allows us to provide an overview of the volume and the focus of available literature, examine how research has been conducted, identify key characteristics, and identify knowledge gaps (38, 42).

### 2.1 Eligibility criteria

According to the recommendations of JBI Manual of Evidence Synthesis (Chapter 11, Scoping Reviews) (42), population, concept and context of interest were defined. This review focusses on the population of adults with chronic primary musculoskeletal pain as a main condition. The concept of study is the investigation of AP with triaxial accelerometry. The context of study is physical activity in daily life. All analytical methods were included if AP were involved, with emphasis on the temporal structure of activity vs. sedentary behavior.

Full-text publications were included with (1) populations over 18 years, (2) primary musculoskeletal CP, (3) AP as a primary outcome measure, (4) triaxial accelerometers and (5) at least five consecutive days of physical activity measurement. In line with the definition of primary CP, publications were excluded if the sample consisted of individuals with musculoskeletal CP resulting from identifiable underlying conditions. Primary CP was selected as the focus of this review because it emphasizes the interaction between pain and AP, rather than the interaction between physical impairments and AP, as seen in conditions such as M. Parkinson or severe osteoarthritis. With mixed samples, papers were included when at least 50% of the sample fulfilled the second inclusion criterium. When subgroups were investigated separately, only data pertaining to the subgroup that met the inclusion criteria were considered.

The minimum of five consecutive measurement days is based on the recommendations of Verbunt et al. (43) for assessing physical activity in CP, as well as prior reliability studies in the general population (44), older adults (45), and individuals with rheumatoid arthritis (46). Eligible publications included peer-reviewed articles, gray literature and dissertations, provided they were written in English, German, Dutch, or French.

### 2.2 Search strategy

PubMed, Embase and CINAHL databases were searched from inception until November 2024. For gray literature the first ten pages of Google Scholar were scanned and dissertations were searched in ProQuest via PsycInfo. The search strategies were developed in cooperation with a specialized information specialist (TE). The search strings for PubMed, Embase, CINAHL, GoogleScholar and PsycInfo were composed by TE. The search strings are included in Supplementary Appendix A. Search was restricted to title, abstract and MeSh terms and included CP, physical behavior, accelerometry and synonyms. Synonyms were based on keyword, MeSh terms (PubMed) and Emtree terms (Embase) of relevant publications. The PubMed, Embase and CINAHL searches were performed by TE. Dissertations were searched by AD in consultation with TE. Finally, references of the included articles were scanned for missed publications.

Six key references (47–52) were selected in advance and in the search results it was verified that the six pre-selected key publications were included.

### 2.3 Processing of search results and selection

References found in PubMed were removed from the Embase and CINAHL results by subtracting PMID-numbers. The outcome was deduplicated in RefWorks Legacy via the close deduplication method and this was double-checked with SR-accelerator Deduplicator.

Selection on inclusion criteria was done in two steps, with (1) a screening phase and (2) a selection phase. Both phases were performed by AD and HW independently with all search results. Inclusions after both screening and selection were compared, discrepancies were discussed, and decisions were made. A third researcher (MV) could be consulted in case of persistent doubt.

For the screening phase title and abstract of all references were imported into Active Learning for Systematic Reviews (ASReview) Lab software (53). With ASReview the screening phase was assisted with an AI-approach, namely Active Learning with different Machine Learning (ML)-algorithms. With this approach, the number of references that need to be manually labeled as relevant or irrelevant is reduced by approximately 90 percent, while maintaining or even improving reliability (54). The active learning method (the ‘ASReview Pipeline’) is summarized in Box 1 and is extensively elucidated by Van der Schoot et al. (53) and Boetje and Van der Schoot (54). The 6 preselected key references were used to check the results of the ASReview-assisted screening phase.

For step 1, ‘adding prior knowledge for training’, AD and HW independently screened and labelled 100 references manually and compared, discussed and adjusted the selection. Steps 2 and 3 were performed by AD and HW independently as well. The resulting two shortlists of included references were compared, and discrepancies were discussed which resulted in a final shortlist. These papers were read full-text and accordance with all inclusion criteria led to final inclusion for this scoping review.

As a final step titles of all reference lists of the included papers were screened independently by HW and AD. The selection phase was similar as with the other databases.

Box 1 **Active Learning for Systematic Reviews, the ASReview pipeline**

Step 1: Prior knowledge is added for training the ML model by manually screening and labelling the first 100 references as ‘include’ or ‘exclude’. With this prior knowledge the ML learning classifier Term Frequency Inverted Document Frequency (TF IDF) with Naive Bayes is trained to predict study relevance. This results in a ranking of all references in the order of relevance.

Step 2: In the active learning part the references are manually labelled as relevant or irrelevant one by one. Each decision is used to train the ML model after which the ranking of relevance is adjusted and a new reference is presented. Decision rules are available to decide whether sufficient references have been assessed to yield a reliable ranking: when (1) all key references are selected (2) at least twice the expected number of relevant references has been screened (3) at least 10% of the total dataset has been screened and (4) screening of at least 50 successive records does not reveal new relevant records.

Step 3: The resulting labelling and ranking of references are used to train the deep learning model Sentence BERT with Fully Connected Neural Network (FCNN). Applying this model results in another ranking of relevance. After this ranking unlabeled records are presented one by one in the order of relevance and judged manually until 50 successive records are labeled as irrelevant.

### 2.4 Data extraction

The study characteristics were extracted from the included articles by the researchers (AD and HW), along with the information necessary to understand the process of methodical reasoning for the quantification of AP. This process involved the successive steps of conceptualizing and operationalizing the concept, followed by selecting measurement properties and data processing.

To find evidence for the existence of concepts or associations with a concept, the concept must be clearly stated and quantifiably defined. The operational definition should include information on the variables (the properties or characteristics) of the concept and its indicators (the methods of quantifying the variables).

**Figure 1:**
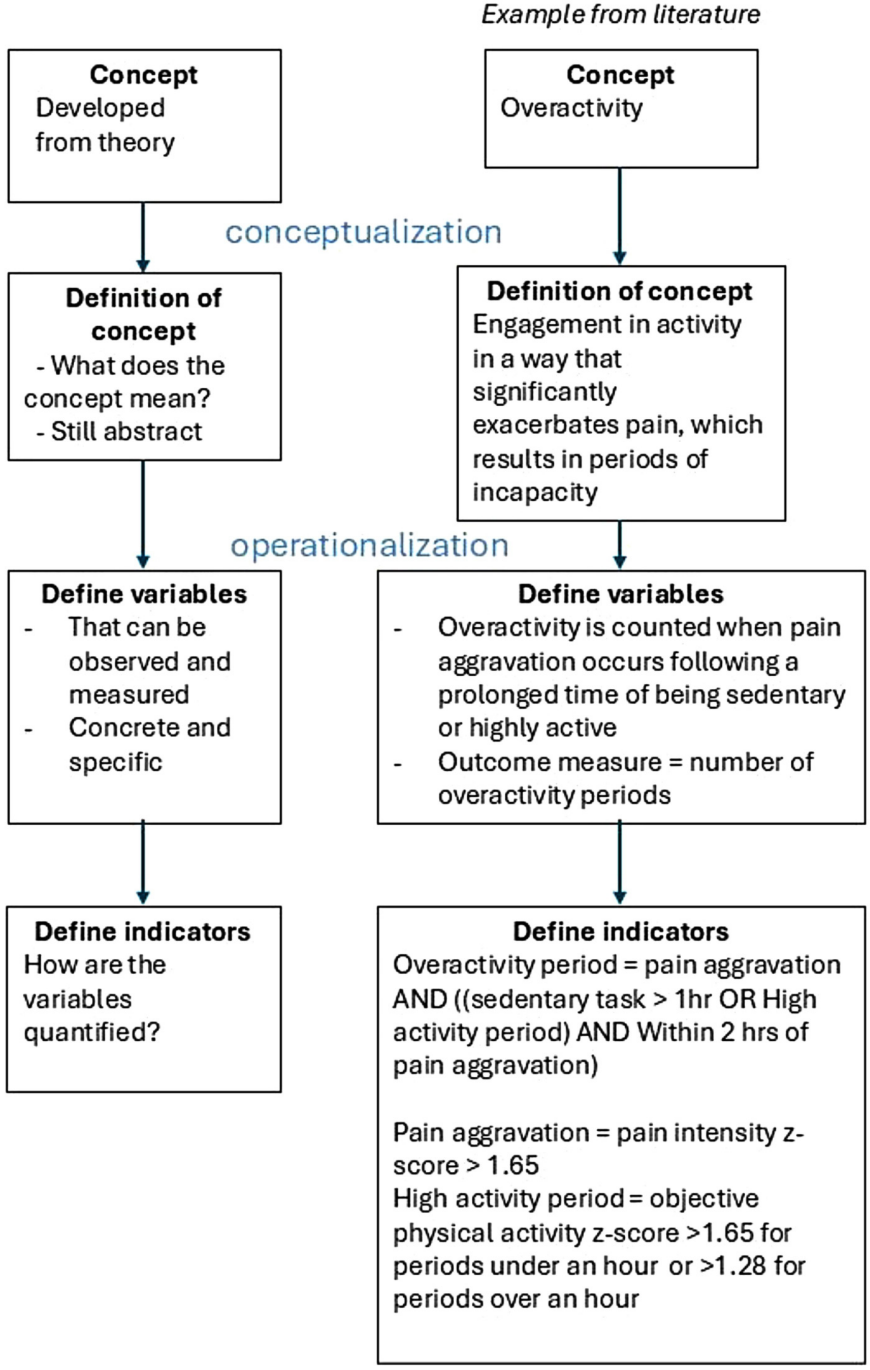
Flow chart of the process of conceptualization and operationalization of a concept, and an example from one of the included papers in this review (51).

This process of conceptualization and operationalization was extracted from the included papers and consisted of four successive steps: (1) formulating the theoretical concept of the study related to AP within CP, (2) defining the concept as precisely as possible (conceptualization), (3) operationalizing the concept with concrete and specific variables, which are the properties and characteristics of the concept, and (4) identifying the concomitant indicators, which quantify variables. This process is clarified by an example in Figure 1. Additionally, measurement properties and data processing were summarized.Lastly, based on this overview of conceptualization, operationalization and data processing, content validity of the included studies will be judged. Content validity is assumed the most important measurement property of a measurement instrument (55, 56), and is defined as the degree to which the content of a measurement instrument reflects the intended outcome being measured (57). When content validity is limited, the value of the conclusions drawn is doubtful.

Content validity encompasses three aspects: (1) relevance for the outcome, target population and context, (2) comprehensiveness, which reflects whether the method includes all necessary components and (3) comprehensibility, which is ‘the extent to which the content of an instrument is understood by the individuals involved in the measurement process, in a manner that aligns with the intention of the developers.’ (55) These measurement properties were not assessed within the included papers, but relevance, comprehensiveness and comprehensibility can be qualitatively assessed by the reader, based on information of conceptualization and operationalization.

## 3 Results

**Figure 2:**
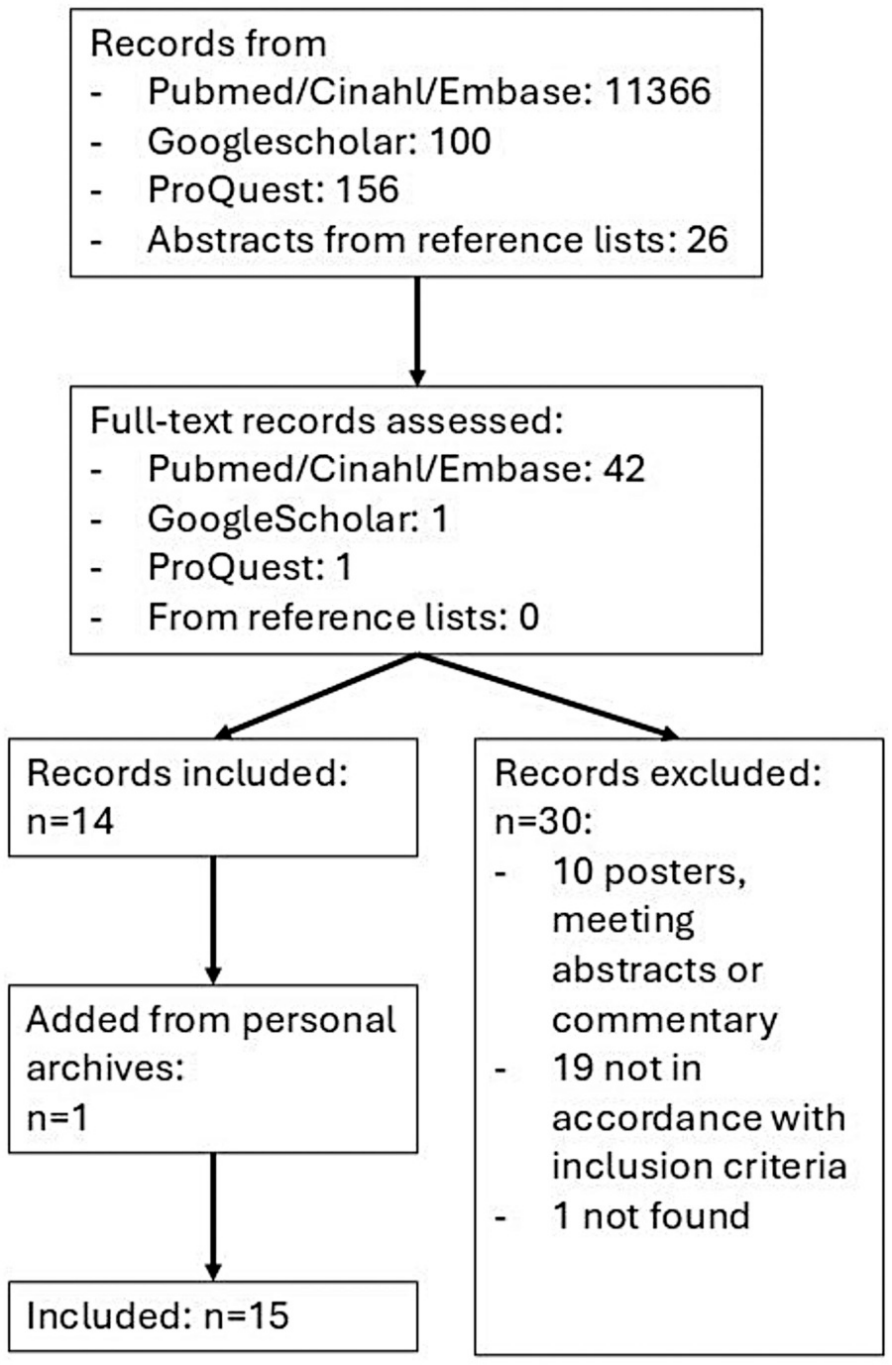
Flow chart of the selection of papers.

As illustrated in Figure 2, after deduplication, the search in PubMed, CINAHL and Embase yielded 11,366 records. With ranking and labelling according to the ASReview pipeline, in total 2,300 of 11,366 references were screened and labelled by HW and AD (in step 1 100 by HW and 100 by AD, in step 2 2 times 1,000, and in step 3 2 times 50). Forty-two references were labelled as relevant, which included all 6 key references. These 42 papers were read full text and inventoried on the inclusion criteria after which 14 papers were included. Ten publications were excluded because they were posters, meeting abstracts or commentaries instead of full text peer reviewed papers. 17 papers were excluded because they did not meet at least one of the inclusion criteria, of which 16 did not investigate AP according to the definition of ‘the temporal structure of physical activity and sedentary behavior accumulated over a specified time period during waking hours,’ and one had a measurement period of one day for investigating activity variability during the day. Of these 17 excluded papers one used a biaxial accelerometer instead of triaxial. In one paper (58) the number of accelerometer-axes was not specified, and, on inquiry, the author could not clarify the number of axes. Paraschiv et al. (25) used a biaxial and a uniaxial accelerometer on different body locations and Paraschiv et al. (59) used three biaxial accelerometers which was assumed to deliver at least the same dimensionality and order of detail as one triaxial accelerometer. Therefore, these two studies by Paraschiv et al. were included. One publication could not be found.

With Google Scholar, 14 papers were ranked as relevant of which 13 could be excluded by reading title and abstract. One publication was selected for full text reading and was excluded because it did not investigate AP. Screening of the titles of ProQuest results yielded 6 records for reading the abstract after which one dissertation was screened full text (60). This dissertation was excluded based on the inclusion criteria. After scanning the reference lists of the 14 included papers 26 titles and abstracts were screened and all were excluded because they did not meet the inclusion criteria. One publication (61) that was not found in the databases was added from the personal archives, which yielded 15 papers for this scoping review.

### 3.1 Study characteristics

The included studies are described in Table 1. Most studies had a cross-sectional set-up. Three studies had a longitudinal set up to investigate treatment outcomes (52), associations between changed behavior-type and disability and quality of life (49) or associations between changed activity level and changed pain intensity (62). CP sample size ranged from 15 to 292. Some studies included a sample without pain (25, 59, 63) or with acute pain (62) for comparison. Most participants were recruited from multidisciplinary pain centers and hospital departments of rehabilitation. In two studies participants were recruited from the general population through their physician (64, 65) and one study recruited participants with advertisement on a university campus (66). In most studies women were predominant and age ranged from 20 to over 74 years (SD 8). In most studies the type of CP was not specified. We assumed that CP in tertiary multidisciplinary pain centers, as in Andrews et al. (51, 67), Liszka-Hackzell et al., Paraschiv *et al*. predominantly comprised primary musculoskeletal CP. In Fanning et al. inclusion criteria were CP in at least two sites of neck, shoulder, back, hip or knee, without specification on the cause of pain. We assumed that this pain mainly comprised chronic primary musculoskeletal pain.

**Table 1:** Characteristics of the included papers.

Author (year)	Study design	Aim of the study related to AP	Sample size	Sample description	Sex (female/male)	Age (sd), range
Andrews (2014) (67)	Cross-sectional	(part of the research) Associations of overactivity with sleep	50	CP from MPC, outpatient, persistent non-cancer at least 3 months, generalized pain affecting gross movement, English literate, >18 years, exclusion = sleep disorder.	30/20	53.22 (10.68), 33–73
Andrews (2015) (51)	Cross-sectional	Associations of objective overactivity with self-report overactivity and avoidance	68	CP from MPC, non-cancer, generalized distribution with impact on gross movement.	44/24	52.85, (11.40), 25–73
Andrews (2023) (52)	Longitudinal cohort baseline, week 7 and week 13	Differences in pacing and avoidance pre- and post- treatment	20	CP from tertiary MPC, selection on overactivity behavior with difficulty implementing pacing strategies and activity-related exacerbations.	9/11	46.9, (NR), 20–67
Fanning (2023) (65)	Longitudinal cohort, Baseline and week 12	Associations of time spent in and bout lengths of different stepping intensities with pain intensity and interference. Change after 12 weeks behavioral program on PA or 12-week control group.	41	CP at least at two sites of neck, shoulder, back, hip or knee, included by physician for online telecoaching and mHealth intervention, BMI 30–45 kg/m^2^, self-reported to be low active, weight stable and no contraindications for exercise.	30/11	69.61 (6.48)
Fanning (2024) (64)	Longitudinal cohort, Baseline and week 12	Associations of stepping patterns with pain and QoL. Change after 12 weeks behavioral program on weight loss and PA.	68	CP at least at two sites of neck, shoulder, back, hip or knee, included by physician for online telecoaching and mHealth intervention, BMI 30–45 kg/m^2^, self-reported to be low active, weight stable and no contraindications for exercise.	52/16	69.53 (6.74)
Huijnen (2011_1) (48)	Cross-sectional	Differences between avoiders, persisters, mixed performers and healthy performers as classified by POAM-P questionnaire Associations of pain intensity with objective activity in avoiders and persisters	116	CLBP from MPC, HDR and via advertisement, 18–65, no specific pathology, no psychiatric disease, no pregnancy	36/43	Avoiders 45.7 (9.8), persisters 48.2 (8.4), mixed 44.2 (10.9), functional 50.6 (12.5)
Huijnen (2011_2) (49)	Longitudinal cohort, baseline, 6 months	Associations between self-discrepancy type and objective avoidance and persistence. Change over time.	116	CLBP from MPC, HDR and via advertisement, 18–65, no specific pathology, no psychiatric disease, no pregnancy	T1 39/45, T2 23/26	47.5 (10.5), 47.8 (10.9)
Huijnen (2020) (47)	Cross-sectional	Differences in objective avoidance and persistence between patients classified by their treating consultant as avoider or persister	16	CLBP from MPC and HDR (>6 months, 18–65), no specific pathology, no pregnancy, no pacemaker, no serious psychiatric disorder	8/8	Avoiders 50 37.5–55.0, persisters 46 45.0–59.0
Liszka-Hackzell (2004) (62)	Longitudinal cohort 3 weeks continuously	Differences between subgroups of chronic and acute pain	15 CLBP, 15 acute pain	CLBP from MPC, pain >6 months. 18–75yrs, (acute <2wks)	CLBP 7/8 Acute 6/9	CLBP 51 (10.2) Acute 46 (10.6)
Neikrug (2017) (61)	Cross-sectional	Associations of activity rhythm parameters with FMS symptoms	292	Fibromyalgia from MPC, community physicians and advertisement	272/20	45.1 (11.1), 21–65
Paraschiv (2008) (25)	Cross-sectional	Differences of dynamics of human activity between CP and no pain.	15 CP, 15 no pain	patients from MPC who are candidate for SCS	7/8	66 (14)
Paraschiv (2012) (59)	Cross-sectional	Associations of dynamics of physical activity with categorized pain intensity (mild, moderate, severe) and age	60 CP, 15 no pain	Patients from MPC who are candidate for SCS	18/42	No pain: 57 (14), severe pain middle age: 54 (9), moderate pain old age: 71 (14), severe pain old age: 74 (8)
Paraschiv (2016) (63)	Cross-sectional	Differences of parameters quantifying the multidimensionality of physical behavior between subgroups mild pain and moderate to severe pain	74 CP, 18 no pain	74 CP patients with chronic intractable pain and candidate for SCS	48/44	63 (14)
Sarwar (2022) (66)	Cross-sectional	Develop machine learning algorithm to predict pain, pain intensity, pain interference and disability from cumulative and relative activity measures, sleep measures and rest activity rhythm measures. Associations between rhythm measures and pain intensity, interference and disability	25 CP, 27 no pain	25 CP mixed	10/12, (3 sex not specified)	NR
Zheng (2023) (50)	Cross-sectional	Differences of physical activity intensity patterns between subgroups in CLBP with high and low central sensitization. Differences with conventional cut-point approach.	42	Primary CLBP from MPC	27/15	39.6 (12.6)

CP, chronic pain; MPC, multidisciplinary pain center; HDR, hospital department of rehabilitation; CLBP, chronic low back pain; PA, physical activity; SCS, spinal cord stimulation; NR, not reported.

### 3.2 Concepts of investigation, definitions, variables and indicators

In most papers a quantifiable definition of the concept of study was available or could be derived from reasoning. As a next step, operational definitions should describe how the concept of study was measured and how the measurements were interpreted. Concepts, their definitions and the definition of variables and their indicators are summarized in Table 2.

**Table 2:** Concepts, definitions of concepts and their operationalization with variables and indicators.

Author (year)	Concept related to activity patterns	Definition of concept (conceptualization)	Definition of variables (operationalization)	Indicators for variables
Andrews (2014)	(Part of the research) Overactivity	Overactivity: high levels of activity → severe pain aggravation + inactivity → sawtooth activity pattern with pain and activity fluctuating greatly over time	Sawtooth pattern = higher fluctuation value of timeseries of vector magnitude of activity counts per minute	Fluctuation value = Root mean square of difference of 2 successive cumulative 15 min vector magnitude
Andrews (2015)	Overactivity	Overactivity: being active in a way that significantly exacerbates pain → periods of incapacity	Level of overactivity = number of overactive periods An overactive period is counted when pain aggravation occurs following a prolonged time of being sedentary or highly active	Overactive period = pain aggravation (pain intensity z-score >1.65) AND sedentary task >1 hr OR High activity period (objective physical activity z-score >1.65) for periods under an hour or >1.28 for periods over an hour. Within 2 h of pain aggravation
Andrews (2023)	Pacing overactivity	Overactivity: too long on an activity (being active or sedentary with sustained spinal position) → pain aggravation Pacing: decreased frequency of overactivity periods	As Andrews (2015)	As Andrews (2015)
Fanning (2023)	The pattern of PA accumulation (i.e., bout length) and rest accumulation	The pattern is described as 1. the time spent in light and moderate physical activity and in rest and 2. the breaks within light and moderate physical activity and rest Hypothesis: Greater sedentary time, fewer sedentary breaks, and sustained participation in more intensive activity will result in worse pain outcome	Activity intensity equals Steps/minute Time spent in rest = Minutes/day being low-active in a seated or lying posture Time being active = Average daily steps and time stepping at different intensities Number of sedentary breaks = Postural shifts from sit to stand Breaks within activity intensities = count of bouts of a certain length (<1 min, 1–5 min, 5–10 min, 10–20 min and >20 min) for each activity intensity	Activity intensity: Moderate: 100–125 steps/min Light: 75–100 steps/min Very light: <75 steps/min Rest: minutes classified by software as low-active in seated or lying position
Fanning (2024)	The pattern of PA intensity throughout the day	PA intensity equals stepping frequency (steps/minute) The PA pattern per participant can be expressed by Fourier functions It is hypothesized that patterns will differentiate in timing of activity and rest and in amplitude	To summarize the PA pattern per participant, a 9-basis Fourier function is derived from each timeseries of steps/minute	Two different types of Fourier functions were distinguished with functional Principal Component Analysis: 1. Amount of stepping 2. Early vs. late risers
Huijnen (2011_1)	Avoidance Persistence	Avoidance: try to escape from activities that are expected to increase pain or injury → low activity levels Persistence: continue activities despite pain until completion → increasing pain → forced rest → sawtooth pattern + longer daily uptime because of postponed rest	1. Persistence = higher physical activity level, more fluctuations, longer daily uptime than avoiders. 2. Persistence = increased pain after increased activity	1a. Daily uptime = wear time. 1b. Mean total activity score = mean counts per day from raw data 1c. Highest activity score = 80% power of highest activity score of monitoring period 1d. Fluctuation score = sum of activity counts during 15 min, then root mean square of difference of 2 subsequent 15 min-periods 2. Increased pain after activity = association between pain and activity level over time with two level hierarchical linear regression analysis
Huijnen (2011_2)	Avoidance Persistence	Not mentioned, but as Huijnen 2011_1 (oral comment)	Persistence = higher scores on daily uptime and activity related style than avoidance	Daily uptime as in Huijnen 2011_1. Activity related style is linear combination of daily uptime, mean total activity score, fluctuation score as in Huijnen 2011_1
Huijnen (2020)	Avoidance Persistence	Avoidance: catastrophizing thoughts about pain + fear of movement → lower daily activity levels Persistence: doing too much, not respecting one's physical limits and experiencing a rebound effect of over-activity → activity levels similar to people without pain	Avoiders will differ from persisters in 1. Overall daily activity level 2. Duration of being active vs. sedentary, 3. Mean general motility (as a measure of intensity; m/s2), and walking motility 4. Number of transitions, and/or 5. Distribution of active vs. sedentary behavior	Distribution measures: 1. Number of active and sedentary bouts, i.e., periods classified as standing, walking, running, cycling or non-cyclic movements vs. sitting or lying 2. Median bout length of active and sedentary behavior 3. Covariance of variation of bout length 4. Fragmentation: number of bouts of physical activity or sedentary divided by total duration of activity or sedentary. 5. W-index for activity or sedentary behavior = (total time of bout lengths above median bout length)/total duration.
Liszka-Hackzell (2004)	Activity-related pain	Increased activity → increased pain (with acute pain, not with CP)	Cross-correlation between pain level and activity counts per minute with time lag up to 60 min	Cross-correlation at different time lags of interpolated pain levels and activity level time series resampled to one sample every 10 min, with time-lags up to 60 min
Neikrug (2017)	Activity rhythms in fibromyalgia syndrome (FMS)	Activity rhythms factor in activity level, timing and duration over multiple days	Activity rhythm parameters: 1. Mesor, 2. Amplitude, 3. Phi, averaged over measurement period And the daily variation (standard error) of these 3 parameters compared to weekly average	1. Mesor = mean activity level in units of the actigraph 2. Amplitude = distance between mesor and peak of curve, according to fitted 24-hr cosine model 3. Phi = time of day of the average peak activity over the week
Paraschiv (2008)	Dynamics of human activity	Dynamics of human activity captured by timeseries of: Sequence of postures Timing Time spent in a posture Any combination	The temporal pattern of each timeseries is quantified with fractal analysis and symbolic dynamic statistics 4 time series: 1. Sequence of posture allocation. 2. Duration of walking periods. 3. Timing of activity-rest transitions as point process. 4. Context dependent symbolic description of the sequence of successive activity-rest periods.	1. Detrended Fluctuation Analysis (DFA) on categorical time series of posture allocation of 4 classified postures (lying, sitting, standing, walking) 2. Cumulative Distribution Function and DFA on sequence of walking episodes characterized by their duration 3. Fano Factor Analysis on time series of the moment in time of transitions from rest (sitting and lying) to activity (standing and walking) and v.v. 4. Symbolic dynamics statistics on symbol series created by coding the comparison of the duration of each activity period with the rest periods just before and after. Values are 0 or 1, 0 = rest period equals activity period. Constructing word sequences from the symbol series
Paraschiv (2012)	Dynamics of sequences of various physical activity states	Dynamics of states are related to structural complexity. Structural complexity depends on the variety of physical activity states and their occurrence in time	Structural complexity: metrics from timeseries of physical activity states: 2 states of lying/sitting dependent on acceleration, 4 states of standing dependent on acceleration, 11 states of walking dependent on cadence and duration	Metrics are determined from timeseries of 18 possible physical activity states, variety of states, temporal structure of state-sequence 1. Complexity metrics: information entropy, Lempel-Ziv complexity and sample entropy 2. Quantitative global metrics: time% spent walking and/or standing 3. Composite deterministic score: sum of the three normalized complexity scores * time% being active 4. Composite statistical score with linear discrimination analysis
Paraschiv (2016)	Multidimensionality of physical behavior	Individual physical behavior: Multidimensional attributes (like type, intensity and duration of activities, movements and postures) Dynamic attributes (the change over time) Relational attributes (factors that modulate behavioral patterns)	Multidimensionality = composite score from metrics quantifying Type Duration Intensity Temporal pattern	Composite score from factor analysis with metrics: 1. % of time walking, % of time on feet 2. 0.975th upper quartile of bout lengths of being active 3. Excess rest vs. deficit rest by plotting cumulative distribution of excess and deficit rest and calculate Kolmogorov–Smirnov distance 4. three types of entropy on timeseries of 18 different states described in Paraschiv 2012
Sarwar (2022)	Rest-activity circadian rhythm	Rest-activity rhythm is quantified by Parameters derived from a fitted cosine curve Intradaily (hour to hour) variability (IV) as a measure of circadian disturbance. IV = the change of activity level from hour to hour. Higher IV indicates more daytime napping or nighttime arousal	Rhythm is quantified with 1. Eight rhythmic features from a cosine curve fitted to 24 h timeseries of activity counts and 2. Intradaily variability of hour-to-hour activity counts	Eight rhythmic features from a cosine curve that is fitted to a 24 h timeseries of activity counts: 1. Mesor 2. Acrophase 3. Amplitude, 4. Relative amplitude = amplitude/mesor 5. Multi-scale entropy (pearson's sample entropy), 6. Mean activity during the most active 10 h (M10, as an estimate of daily activity), 7. Mean activity during the least active 5 h (L5, as an estimate of nocturnal activity), 8. rest-activity relative amplitude ((M10-L5)/(M10 + L5)), Intradaily variability: $IV=\frac{N\sum_{i=2}^{N}(x_{i}{-}_{i-1}^{2})}{(N-1)\sum_{i=1}^{N}{\left(x_{i}-\mu \right)}^{2}}$ Where: *N* is the total number of datapoints, *x_i_* are the individual data points and μ is their mean
Zheng.(2023)	PA intensity patterns	PA intensity patterns: Temporal organization of PA intensity levels Transition between PA intensity levels	Pattern: Bout duration of 5 hidden states reflecting 5 intensity levels Accumulated time per hidden state per day Transition probability from one hidden state to every other hidden state Hidden states: Derived from accelerometer time series with a machine learning algorithm Reflect 5 intensity classes	Pattern = One value of bout duration per intensity class One value of accumulated time per intensity Values for transition probability from each intensity to each other intensity

PA, physical activity.

#### 3.2.1 Concepts and definitions (conceptualization)

The concepts used to investigate AP were diverse (Table 2), and the meaning of these concepts could be extracted from all papers. Definitions and specifications of these concepts are presented in Table 2. A distinction could be made between concepts related to behavioral patterns and those derived from physics.

Six papers (47–49, 51, 52, 67) utilized existing models of behavioral patterns related to activity and rest as their research concept, analyzing accelerometer time series, sometimes combined with pain intensity time series.

The definition of overactivity in Andrews et al. (67) was in line with the definition of persistence in Huijnen et al. (48, 49). Andrews et al. (51, 52) expanded this definition to include pain aggravation after prolonged sedentary periods. Huijnen et al. (47), changed the definition of persistence to ‘doing too much, not respecting one's physical limits and experiencing a rebound effect of over-activity’ Andrews et al. introduced the concept of pacing, defined as a lower frequency of overactivity.

Time series of pain were included in four of the six papers on persistence and overactivity due to the hypothesized relationship between pain and activity in these behavioral patterns. Andrews et al. and Huijnen et al. (49) did not include pain levels, focusing on fluctuation values of physical activity intensities, daily uptime and mean activity Huijnen *et al*.

The remaining nine papers used physics derived measures to capture temporal patterns or complexity: (1) fitted Fourier functions (64), (2) the pattern of activity and rest accumulation (65), (3) the causal relation between activity level and pain Liszka-Hackzell *et al*. (62), (4) the rhythmicity of rest and activity during the day with fitted cosine curves (61, 66), (5) the dynamics of human activity (25), more clearly defined in Paraschiv et al. (59) as the temporal and dynamical structure of human physical activity, and adopted in Paraschiv et al. (63). The three Paraschiv-papers build on the method of constructing time series of activity type and intensity of walking derived from accelerometry as presented in Paraschiv *et al*. (68). Lastly, Zheng et al. (50) used the temporal organization of activity intensity levels combined with transitions between intensity levels to investigate AP.

#### 3.2.2 Variables and indicators (operationalization)

In all the papers, observable and measurable variables for operationalizing the concept of study were defined, and descriptions of the procedures for quantifying these variables were included.

**Figure 3:**
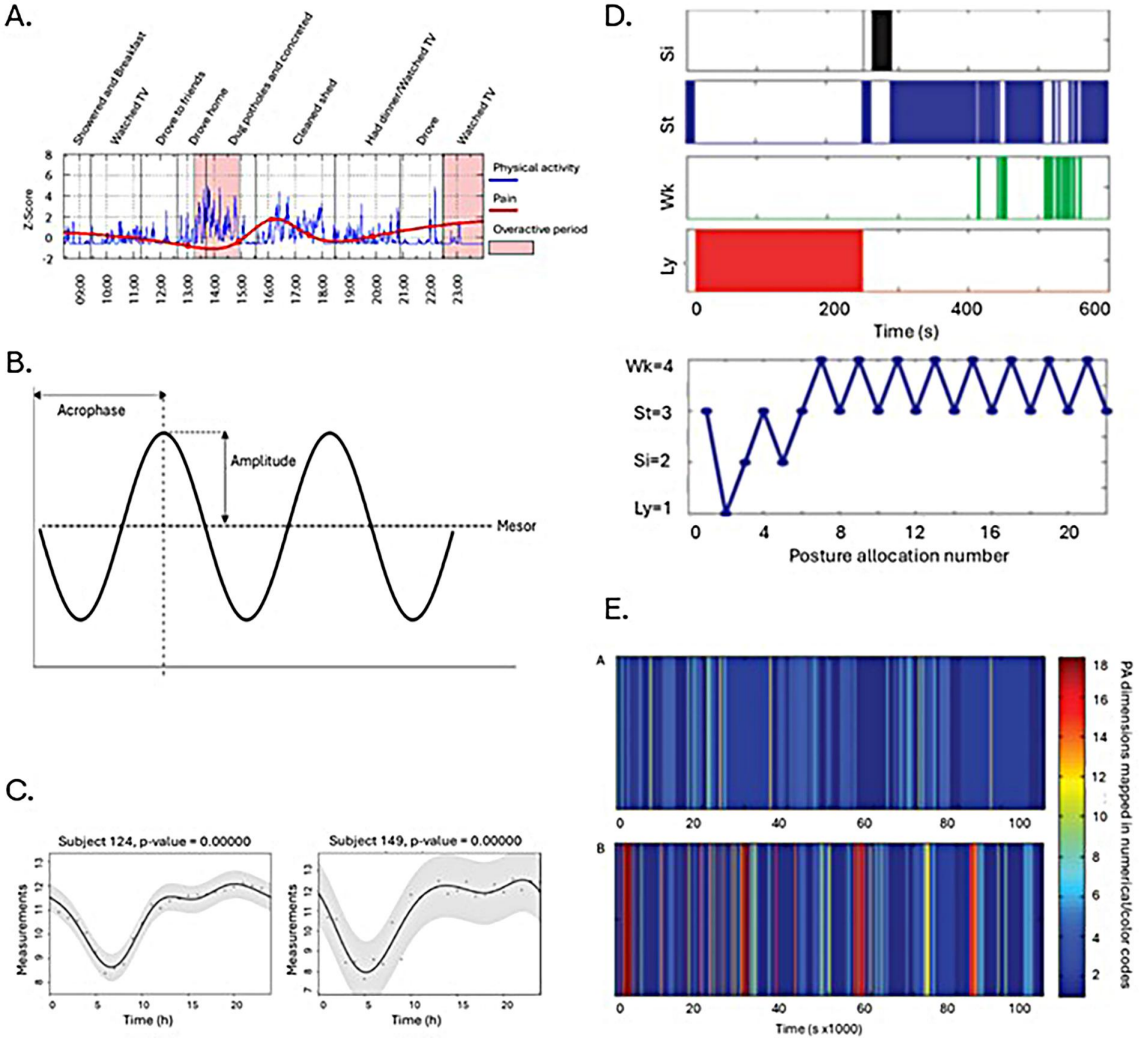
Impression of the variation of methods used to extract activity patterns from accelerometer time series across studies: **(A)** overactivity periods (52), **(B)** rhythm parameters (66), **(C)** with cosinor fitting (66), **(D)** posture allocation (25), and **(E)** physical activity states (59).

In the six papers that used behavioral concepts (47–49, 51, 52, 67), variables were formulated to inventory the occurrence or nonoccurrence of the behavior. Due to the expected sawtooth pattern with overactivity, Andrews et al. focused on the fluctuation of activity levels. In Andrews et al. (51, 52), the outcome measures emphasized the concomitant pain increase with overactivity (Figure 3A), rather than focusing on the fluctuation values related to sawtooth pattern. Huijnen et al. (48) compared variables related to avoidance and persistence between persons classified as avoider or persister with the POAM-P self-report questionnaire. Persisters were expected to have a longer daily uptime, a higher average activity level and more fluctuations in activity level compared to avoiders. Additionally, it was hypothesized that in persisters, the time series of activity level and pain are associated.

To quantify the level of persistence, Huijnen et al. (49) calculated the daily uptime and a linear composite score of daily uptime, mean total activity score and a fluctuation score. To investigate differences in activity behavior between individuals classified as avoider, persister, mixed performer or healthy performer, Huijnen et al. (47) used cumulative variables such as overall daily activity level and total sedentary time, as well as variables representing the distribution of activity and rest over time.

In Fanning et al. (65) the concept of accumulation of rest and physical activity was operationalized using a combination of cumulative measures and breaks within bouts of rest and activity. To investigate the timing of activity and rest as well as the amplitude of activity intensity, Fanning et al. (64) fitted a 9-basis Fourier-function on each participant's time series of steps per minute. Subsequently, they performed a functional Principal Component Analysis to identify a set of Fourier functions capturing the most variability.

Neikrug et al. (61) extracted day to day rhythmic features of activity and rest using cosinor-based techniques and determined correlations with fibromyalgia symptoms. Besides the three parameters in Neikrug et al., Sarwar et al. (66) extracted six other rhythmic features with a cosinor model. Some of these parameters and a fitted cosinor model are shown in Figures 3B,C.

Liszka-Hackzell et al. (62) calculated the cross-correlation of interpolated time series of pain levels with time series of activity counts per minute. Cross-correlation was determined with a time lag of the pain time series of −60, −30, 0, 30 and 60 min, with which they investigated whether a pain level increase was ahead of activity level increase, synchronous, or delayed.

Paraschiv et al. derived different time series types of postures and activity intensity from accelerometer data, refining their methods in successive papers (25, 59, 63), based on the method presented in Paraschiv et al. (68). In the 2004 paper, four different postures (lying, sitting, standing and walking) and the intensity of walking were derived from accelerometer data using discrete wavelet transformation, Savitzky-Golay filters, vector functions and gait analysis parameters. With these methods, Paraschiv et al. constructed time series, including the sequence of posture allocation (Figure 3D), the sequence of the duration of walking episodes, the timing of transitions from rest to activity and vice versa, and the duration of activity relative to the duration of rest before and after activity, represented as a symbolic sequence. Nonlinear analyses were applied to these time series to investigate the AP. Paraschiv et al. added intensity to the four activity types based on different acceleration thresholds for each activity type. This resulted in 18 possible physical activity states. Lastly, to explore the ability of a composite score to characterize physical behavior, Paraschiv et al. performed a factor analysis on outcome variables from these 18 activity states, and assessed the association of pain intensity with activity behavior with multiple regression and discriminant analysis.

Zheng et al. (50) applied unsupervised learning (Hidden Semi-Markow modelling, HSMM) where ML-algorithms were used to discover a set of hidden states in unlabeled accelerometer data. The modelling identified five hidden states corresponding to five different activity levels.

In summary, AP related research concepts were operationalized in many ways. Figure 3 provides an impression of the variation of methods used. Methods included a measure of fluctuation by subtracting two successive bouts of activity intensity, timing and amplitude of activity intensity quantified by fitted Fourier-functions, parameters derived from a fitted cosine curve, non-linear analyses, complexity metrics and variables that quantify fluctuations and distribution like transition frequency and W-index.

### 3.3 Measurement properties and data processing

Measurement properties provide information on how data were collected. Data processing refers to the conversion of raw accelerometer data into the outcome measure needed for further analysis (see Table 3).

**Table 3:** Measurement properties and data processing.

Author (year)	Device	Wear location	Measurement frequency	Duration	Other principal variables for AP-related concept of study	Other variables for associations, differences between groups or treatment results	Valid data definition	Epoch length	Conversion method
Andrews (2014)	GT3X Actigraph	NR	30 Hz	5 days + nights, at least 1 weekend day	Pain intensity 11-point VAS, (mood, stress, catastrophizing) 6x/day	Parameters of sleep derived from accelerometry	NR	1 min	Activity counts per minute and then vector count per minute
Andrews (2015)	GT3X Actigraph	NR	30 Hz	5 days, at least 1 weekend day	Pain intensity 11-point VAS, 6 times/day at random intervals. Diary	Self-reported approach to activity (PARQ)	4 complete days for each parameter	1 min	Activity counts per minute and then vector count per minute
Andrews (2023)	GT3X Actigraph	Waist	NR	5 days	Pain intensity (not specified), 1 /hr, interpolated to 1 /min	Average pain, average activity level, medication intake, self-reported overactivity, avoidance, depression, anxiety, stress and time in leisure, social, rest or productive tasks.	(1) At least 75% of waking hours could be accounted for by diary activities entered in the Pain ROADMAP app and (2) Actigraph data were available for this same time period. At least five of the 7days of monitoring needed to be classified as a valid data collection day for the whole monitoring period to be considered valid	1 min	GT3X automatically converses changes in tri-axial acceleration to activity counts per minute. Vector magnitude from activity counts per minute in 3 axes was calculated
Fanning (2023)	ActivPAL 4	Upper midline of thigh	NR	7 days	None	PROMIS Pain intensity scale, PROMIS Pain interference scale	3 days	NR	Data processing with PALBatch 8.11.63. Classification to stepping, lying and sitting with CREA algorithm 1.3 Ambulatory activity intensity is derived from stepping cadence bands
Fanning (2024)	ActivPAL 4	Upper midline of thigh	NR	7 days	None	PROMIS Pain intensity scale, PROMIS Pain interference scale, Health-related quality of life with SF-36 (physical function, emotional role limitations, physical role limitations, energy/fatigue, emotional well-being, social function, pain and general health)	3 days	1 min	Data processing with PALBatch 8.11.63. Data classification with CREA algorithm 1.3. Both not specified
Huijnen (2011_1)	RT3	NR	NR	14 days	Pain intensity 8× /day 7-point Likert-scale	Classification of participants as avoider, persister, mixed performer or functional performer with POAM-P	At least 5 days including 1 weekend day. 1 valid day has at least 10hrs.	1 min	1. Resultant vector from 3D signal. 2. counts per minute of exceedance of predefined threshold. For association with pain: mean activity signal between two pain measurements
Huijnen (2011_2)	RT3	NR	NR	14 days	None	Self-discrepancy type with HSQ, age, gender, pain duration, mean pain intensity	At least 5 days including 1 weekend day.	1 min	As in Huijnen 2011 (1)
Huijnen (2020)	VitaMove activity monitor	Chest + left and right thigh	128 Hz	5 days	None	Classification of participants as avoider or persister by treating consultant	Number of days flexible.	1 s for analyses of postures, motions and transitions	Detection of postures and motions 1/sec with VitaScore Software
Liszka-Hackzell (2004)	AW-64 actiwatch	Non-dominant arm	NR	3 weeks	Pain 11-point VAS at least every 90 min	Having chronic or acute LBP	At least 14 complete days of activity and pain	1 min	Activity was sampled as accumulated counts/minute
Neikrug (2017)	MicroMini-Motionlogger Actigraph	Non-dominant wrist	32 Hz	7 days	None	Pain severity and interference (MPI), physical impairment and functioning (FIQ), fatigue (MFI), mood (CESD), sleep (from actigraph)	Equal or less than 1 night missing or less than 8 h missing data during the day	1 min	With actigraph Action-3 software, outcome parameter not specified
Paraschiv (2008)	1 biaxial and 1 uniaxial accelerometer ADXL202 + gyroscope	Biaxial chest, uni-axial thigh, gyroscope thigh + shank	40 Hz	5 weekdays, 8 h/day	None	None	NR	1 s	Discrete wavelet transformation, Savitzky-Golay smoothing filters and numerical gradient on raw data. Then: 1. type of activity with previously developed algorithm (68) 2. intensity of walking from mean walking cadence during each walking period. 3. intensity of sitting lying standing with trunk acceleration norm
Paraschiv (2012)	3× biaxial ADXL202 + gyroscope	Sternum, mediolateral thigh, shank	40 Hz	5 weekdays, 8 h/day	None	Pain-score classified as no, moderate and severe pain. Age classified as middle age and old age	NR	1 s	As Paraschiv et al. (2008)
Paraschiv (2016)	2× triaxial MMA7341LT + gyroscope	Sternum and mediolateral axis of thigh	40 Hz	5 weekdays, 8 h/day	None	Pain-score VAS classified as mild pain (VAS≤4) and moderate to severe pain (VAS>4),	NR	1 s	As Paraschiv et al. (2008)
Sarwar (2022)	Actigraph GT3X	Wrist	NR	5 days + nights on weekdays	None	Average pain intensity, pain interference and disability with PROMIS-29 v2.0	Days with at least 20% of complete data were included	1 h	Activity and sleep variables with algorithms from ActiLife software, resampled to 1 h and smoothed with 3 h simple moving average
Zheng (2023)	GT3X	Front right hip (Anterior superior iliac spine)	100 Hz	Approx. 1 week, excl sleeping and bathing	None	Central sensitization symptoms with CSI	Days with complete 24 h covered. 4 days, randomly selected	5 s	Gravity effects removed from raw data, vector magnitude calculated, averaged over 5 s. For comparison with conventional cut points approach: Resampled to 30 Hz, then bandpass Butterworth filter with 4 orders, then filter with coefficient matrices from Brønd (85)

NR, not reported; SF-36, 36-item short-form survey; HSQ, Hardin's selves questionnaire; POAM-P, patterns of activity measure—pain; TSK, tampa scale of kinesiophobia; LBP, low back pain; MPI, multidimensional pain inventory; FIQ, fibromyalgia impact questionnaire; MFI, multidimensional fatigue inventory; CESD, center for the epidemiological studies depression scale; PARQ, physical activity readiness questionnaire; PROMIS-29, patient-reported outcomes measurement information system—29; CSI, central sensitization inventor

Measurement properties were heterogeneous (Table 3). Thirteen papers used triaxial accelerometers and two papers used a combination of multiple biaxial and/or uniaxial accelerometers (25, 59). In two studies the accelerometer was worn on the non-dominant arm (62) or wrist (61) and in one study the side of the wrist-worn accelerometer was not specified (66). In one study the accelerometer was attached to the waist (52), in two studies to the upper midline of the thigh (64, 65), and in another study to the front right hip (50). Five studies used multiple accelerometers on multiple wear locations [chest and both thighs (47), sternum and mediolateral axis of thigh (63), sternum with mediolateral axis of thigh and shank (59) and chest with thigh and shank 25]. In four studies the wear-location was not reported (48, 49, 51, 67).

Measurement frequency ranged from 30 to 128 Hz, with frequencies from 30 to 40 Hz being predominant. Measurement frequency was not reported in seven studies (48, 49, 52, 62, 64–66). Sampling duration ranged from 5 to 21 days, with a duration of five days being predominant. Of the studies that sampled five days, two studies included one weekend day (51, 67), four studies only included weekdays (25, 59, 63, 66) and four studies didn’t specify which weekdays were covered (47, 52, 64, 65). Valid data was defined in most studies. Epoch length ranged from one second to one hour, with one minute being predominant.

Four studies sampled pain intensity during the day and used these time series to investigate AP since pain was part of the definition and operationalization of the concept of research (48, 51, 52, 62). Time series of pain were measured with 11-point visual analogue scale (51, 62) or a 7-point likert-scale (48). The pain measurement instrument was not specified in Andrews *et al*. Many other variables were sampled to investigate associations, associations through time and differences between subgroups. These variables include age, sex, self-reported measures of pain intensity, pain duration, pain interference, health-related quality of life, medication intake, approach to activity, depression, anxiety, self-discrepancy type, fatigue, impairment, functioning, mood, sleep and central sensitization symptoms.

Triaxial accelerometry results in time series of acceleration around x-, y- and z- axes, with the number of values per second dependent on sampling frequency. These time series were converted to another parameter in most of the included papers. In general, the description of conversion methods was limited. Some authors only refer to software packages and some to manuals or websites of the accelerometer manufacturer that were no longer available on the web.

For data processing six studies transformed the acceleration time series to activity counts per minute, also named vector counts per minute (48, 49, 51, 52, 62, 67). This method operates on the assumption that counts per minute is associated with the energy expenditure of activities and therefore with activity intensity. The vector magnitude of acceleration was calculated from the triaxial acceleration values and to yield counts per minute the number of times per minute of exceedance of a predefined threshold value was counted. The threshold for a count was not specified in the papers. Two studies transformed the acceleration time series to steps per minute and postures of lying, sitting or standing (64, 65).

Paraschiv *et al*. (25, 59, 63) processed accelerometer data with discrete wavelet transformation, Savitzky-Golay filters and a numerical gradient. Subsequently they constructed time series of activity type with a previously developed algorithm (68). They used different methods to detect different activities, postures and intensity of walking. Zheng et al. (50) used raw accelerometer data from which the gravity effects were removed and then computed vector magnitude. Unsupervised learning was applied to these time series which resulted in time series of five activity intensity classes. For comparison of result with a traditional method, they applied the cut-off points approach as well. With this approach the tri-axial acceleration signal was converted to vector magnitude and thresholds were defined for different activity levels.

### 3.4 Validity of conceptualization and operationalization

Content validity was assessed to determine the extent to which the operationalization accurately reflects the definitions of the concepts. Content validity encompasses three aspects: relevance, comprehensiveness and comprehensibility.

The summary of conceptualization and operationalization, as illustrated in Table 2, reveals that content validity is sufficient in some studies, but limited in others. For example, Andrews et al. (67) operationalize the concept of overactivity using a fluctuation value. However, fluctuating activity levels may be normal or even adaptive. To identify maladaptive fluctuations in the context of overactivity associated with pain, it is essential to include at least the correlation of pain with activity intensity. This was addressed in subsequent studies of Andrews et al. (51, 52), although these papers do not quantify the period of incapacity resulting from the rebound effect of excessive activity.

Therefore, these studies do not fully capture all dimensions of overactivity as a maladaptive strategy, as defined by the authors. The comprehensiveness of the operationalization can thus be rated as insufficient, raising concerns about whether the outcome variables truly reflect the construct of overactivity. A similar conclusion applies to the operationalization of persistence in Huijnen et al. (48) that includes a similar fluctuation value and the association of pain with activity intensity. The concept of pacing in Andrews et al. (52) is directly linked to the operationalization of the concept of overactivity, as it is defined as a reduced frequency of overactivity. Consequently, the content validity of pacing in this study is also insufficient.

In Huijnen et al. (47) the definitions of avoidance and persistence partly diverged from those used in earlier publications. The definition of avoidance emphasized reduced activity levels due to catastrophizing thoughts and fear of movement. Yet, these motivational aspects—catastrophizing and fear—were not measured, resulting in insufficient comprehensiveness. The concept of persistence was further refined to excessive persistence and defined as doing too much, not respecting one's physical limits and experiencing a rebound effect of over-activity. However, the successive rebound effect of doing too much was not captured in the outcome variables and pain intensity was not included in the analyses, therewith raising similar concerns on content validity as those identified in previous studies by Huijnen *et al*. and Andrews *et al*.

With physics based concepts, achieving comprehensiveness is generally less challenging as the concepts and their definitions are grounded in quantifiable parameters. For example, the extraction of rhythm parameters using cosinor fitting—applied to investigate activity rhythms as demonstrated by Neikrug et al. (61) and Sarwar et al. (66)—is a clearly valid method. However, the studies by Fanning et al. (64, 65) raise concerns regarding the comprehensiveness of AP profiling. In these studies activity intensity classes are derived from timeseries of stepping frequency, which inherently exclude non-stepping activities such as cycling, swimming and seated activities. Consequently, it is questionable whether the constructed time series adequately capture AP.

Some studies with physics based concepts raise doubts on relevance and interpretability. For example the study of Fanning et al. (65) counted the number of bouts of certain lengths for different intensity classes and investigated the change of these numbers of bouts after a 12-week behavioral program. Some change values correlated with pain intensity and pain interference. For example an increase of the number of bouts shorter than 5 min with light activity intensity correlated significantly with an increased pain intensity, but not with pain interference, while a changed number of 5-minute bouts of very light and moderate intensity did not correlate with pain intensity (see Supplementary Appendix B). Although the authors provide some interpretation regarding the association between changes in 5-minute bouts of light intensity activity and pain, the overall interpretation and practical relevance of the combined findings remain ambiguous. This is further compounded by the substantial risk of Type I error, given that associations between 22 parameters and pain and pain interference were examined with a relatively small sample size of 41.

The indicators derived from the variables by Paraschiv et al. (25, 59, 63) are methodologically advanced, yet they may be difficult to interpret by general health care researcher and practitioners, particularly the composite scores. For ensuring their relevance and applicability in daily practice, it is essential to establish the meaning of these indicators by examining their associations with healthcare outcome measures, like state parameters pain, fatigue and mood.

Hypotheses testing may yield further, but indirect, insights into the validity of the measurement instruments by evaluating the consistency of outcome variable scores with predefined hypotheses. This approach assumes that the measurement method validly measures the intended construct. All reviewed studies employed hypotheses testing by examining associations between selected indicators and related behavioral types or clinically relevant outcome parameters, as well as by assessing group differences and intervention-related changes. However, the majority of AP variables showed no significant associations (see Appendix B for a detailed overview of significant and non-significant findings). This lack of associations may reflect inadequacies in the conceptualization and/or operationalization of the concepts, irrelevance of the constructs or outcome variables, or limitations inherent to the comparator instruments—such as insufficient construct overlap or suboptimal clinimetric properties.

Although the number of significant associations between behavioral type and objective AP variables was small (see Supplementary Figure A in Appendix B), the observed associations appeared plausible. For instance, based on established definitions, it is reasonable to expect that persisters exhibit greater fluctuations in activity (51) and longer days (48) compared to avoiders. Notably, Paraschiv et al. (63) was among the few studies that consistently identified associations between pain intensity and objective AP parameters. The consistency reported by Paraschiv et al. (63) may be attributed to the use of more advanced accelerometer data processing techniques and the application of AP composite scores derived through data-driven factor analysis.

## 4 Discussion

With this scoping review we aimed to create an overview of methodical reasoning within studies investigating AP in patients with CP with accelerometers. This methodical reasoning included the consecutive steps of (1) selection of the AP-concept of research, (2) its definition or specification (conceptualization), (3) the definition of variables and indicators that can be observed and measured (operationalization), and (4) choosing measurement properties and methods for data processing and extraction of indicators from raw accelerometer data. With this information we aimed to provide insight into the availability and validity of concepts and measurement methods for appliance in future research.

The research concepts were diverse, encompassing behavioral concepts and physics derived concepts. Behavioral concepts included avoidance, persistence (also known as overactivity), and pacing. Physics derived concepts involved the temporal association of pain with activity, activity rhythms, the multidimensionality of activity behavior, the dynamics of activity, and activity intensity patterns. Behavioral concepts were defined using hypotheses and theories extracted from the literature. Operationalization was achieved through decision rules or parameters reflecting various aspects such as the amount of activity, timing of activity, activity intensity, distribution of different activity intensities, bout length within activity intensity classes, transitions, complexity, variability and the correlation of pain with activity level.

The operationalization of behavioral concepts proved to be challenging, raising concerns regarding comprehensiveness. With physics based concepts, in some cases, relevance and interpretability of outcome variables were unclear.

The difficulty in operationalizing behavioral concepts of avoidance, persistence, overactivity and pacing into quantifiable AP variables is understandable. These behavioral concepts encompass multiple dimensions, including symptoms (e.g., pain intensity), functional status (e.g., disability and pain interference), and characteristics of the individual (e.g., motivation for physical activity and personal values) (69). Since a single measurement instrument is typically designed to assess only one construct, an AP parameter, by definition, cannot fully capture the complexity of behavioral concepts.

In contrast, physics-based concepts benefit from their grounding in quantifiable and objective parameters, which simplifies their operationalization. These constructs are typically derived from well-established measurement techniques, reducing ambiguity in their definition and application. However, despite their methodological robustness, the relevance of physics-based indicators in daily clinical practice is not always evident. In some cases, the interpretability of outcome variables may be limited. It is acknowledged that all studies were exploratory in nature and did not primarily aim to develop measurement methods suitable for clinical application.

The comparison of study results and the interpretation of their collective implications for the usability of the methods are further impeded by the heterogeneity of measurement properties and data processing. This heterogeneity included variations in sensor brand, wear locations, measurement frequencies, durations, epoch lengths, and conversion methods. Moreover, the reporting of measurement properties and data processing methods was incomplete in multiple studies (25, 48, 49, 51, 52, 59, 62–67), while this information is crucial for interpreting, comparing, validating and reproducing results and conclusions.

The diversity of conversion methods resulted in a diversity of outcomes reflecting activity intensity and rest (Table 3). For example, multiple studies converted raw accelerometer data to vector counts per minute and used cut-off points as a measure of activity intensity (11, 48, 51, 52, 62, 67). Two studies converted raw data to steps/minute, and classified this to activity intensities (64, 65). Another set of studies converted raw data to time series of type of activity, intensity of walking, sitting, lying and standing (25, 59, 63). Methods to extract AP variables from these outcomes were even more diverse (Table 2). The clinimetric properties of these different conversion methods are unclear as the papers did not provide information on their validity and reliability.

Previous research and the results within this review provide some information on the reliability of the conversion methods. Multiple studies concluded that cut-off points are protocol-, population- and brand specific (70, 71). While most recent studies deploying accelerometers still use cut-off point approaches (72), Staudenmayer et al. (73) found that ML-models perform better than traditional linear and cut-off points models in estimating activity intensities (73). The improved reliability of a ML model in converting accelerometer data to activity intensity might influence measured associations or differences between groups.

This is evidenced by the studies of Zheng et al. (50) and Paraschiv et al. (63). Zheng et al. found no differences in physical activity between groups of chronic low back pain patients with and without central sensitization using the traditional cut-points approach. However, significant differences were found for five AP parameters using an ML approach. Similarly, Paraschiv et al. consistently found associations between pain intensity and objective AP parameters, which might be explained by the more advanced methods to process accelerometer data and the more sophisticated AP parameters applied. This indicates the added value of advanced methods for data processing and extracting outcome measures in this field of research. A recent scoping review summarized more advanced methods to operationalize the concept of AP from accelerometer data (35).

### 4.1 Recommendations

The clinical significance of objectively measuring AP is considerable. Healthcare professionals working with patients with chronic pain play a crucial role in helping patients to manage their pain and improve their ability to engage in desired activities, daily functioning and participation. Currently, they lack objective information to guide their treatment decisions. In daily practice, it is essential to recognize that the choice of measurement method impacts validity and, consequently, the added value for clinical reasoning.

Future studies investigating AP should use valid, reliable and responsive measurement instruments. Information and tools for selecting health measurement instruments are available with the COSMIN-initiative (56, 74, 75). While these tools are developed for patient reported outcome measurement instruments, the same principles are applicable to performance outcomes like accelerometer outcomes. Moreover, comprehensive reporting of methods is essential to ensure interpretability, comparability, and progress in the research field. Regarding accelerometry, reporting should include wear location, sensor brand and model, sampling frequency, feature extraction method, window size or epoch length, and the number of axes. Uniformity of methods is needed to develop insights into the usefulness and clinimetric properties of AP variables. The current standard for accelerometer data processing is ML. Therefore, measurement properties and data processing should be investigated with this method. As a critical step, a large annotated dataset for training and testing ML-algorithms has been published recently (76).

As this review showed major difficulties with measurement methods based on behavioral concepts, it might be recommended to use data-driven approaches in future research. Data driven methods (like ML, Hidden Markov modelling and Principal Component Analysis) may provide more comprehensive and detailed insights into pattern parameters relevant to daily practice, particularly when these parameters are associated with clinical outcomes. Conversely, while data driven methods can extract AP parameters, their clinical significance may remain ambiguous and thus require interpretation by the researcher.

This review highlighted some potentially valuable AP-parameters including intradaily variability (66), the complexity of the activity intensity signal (59), and amplitude of the activity intensity signal (61). Another recent review provides overview of more advanced analytical methods and variables for assessing physical activity behavior (35). Those variables were classified into three categories: activity intensity distribution, activity accumulation, and temporal correlation and regularity.

Comparing self-report AP-questionnaires with objectively measured AP poses some other caveats, as questionnaires and accelerometers measure different constructs. Self-report questionnaires depend on recalling behavior and measure perceptions, thoughts and feelings, while accelerometers measure actual movement, partly explaining the inconsistent associations found between accelerometry and questionnaires to investigate AP.

In daily practice it is important to recognize that questionnaires and accelerometers measure different concepts and therefore can be complementary. Scores on questionnaires are affected by many patient-specific psychosocial confounders. For example, participants with a more depressed mood tend to rate their behavior more negatively (77), and the perception of one's own behavior is influenced by reference groups. Discrepancies between questionnaires and accelerometer data provide further information on the appreciation of one's own behavior. For instance, an unrealistic negative appreciation is mostly maladaptive, and this information could help in formulating treatment goals and interventions. Notably, hypothesis testing should be conducted only after the validity and reliability of all employed measurement instruments have been rigorously established.

A Delphi-study or workshops might be a first step towards consensus on validity and clinical relevance of methods in future research. It is plausible that the combination of insights and methods from movement sciences, behavioral sciences, physics and data science, and observations from daily practice will yield the most meaningful understanding of valid and relevant methods for investigating AP in CP. Consequently, multidisciplinary research is needed, involving researchers, experts from daily practice and patients.

### 4.2 Strengths and limitations

The strength of this study lies in the structured and detailed extraction of information on the methodical reasoning process within studies measuring AP, therewith providing overview of methods used, and insight in the usability and validity of methods for future research and in comparability of results. This method emphasizes the importance of sound and clean conceptualization and operationalization in this complex field of behavioral research.

This review did not yield sound conclusions on useful and useable methods and parameters for analyzing AP within CP due to the small number of available studies, small sample sizes, the great diversity of measuring and conversion methods, the diversity of outcome measures, incomplete method reporting and concerns on validity.

Although we provided a detailed overview of the research conducted on this topic, certain aspects were not addressed in this review. For valid and reliable measurement and to enable comparison of research, consensus on measurement properties and data processing is needed. This review does not yield substantiated recommendations on these topics. Recommendations should include accelerometer calibration, sampling frequency, epoch length, wear location, the use of filters, the number of days required to obtain a reliable representation of behavior, conversion methods and the validity of algorithms in real-life. While these topics are covered in recent research (35, 78–84), consensus is still lacking.

## 5 Conclusion

This scoping review highlighted the importance of sound and clear methodical reasoning when aiming to measure activity pattern concepts with accelerometers in health care, especially within the context of chronic pain. We conducted this study by systematically addressing the consecutive steps of concept selection, conceptualization, operationalization, and the evaluation of measurement properties and data processing. The diversity of methods and the limited reporting in many cases have hindered the validation of the included studies’ methods and results.

## Data Availability

The original contributions presented in the study are included in the article/supplementary material, further inquiries can be directed to the corresponding author.
