# Supplementary material for: From accelerometer data to activity patterns in chronic pain: methodical reasoning is key
Source: Front Sports Act Living. 2026 Feb 23;8:1712235. doi: 10.3389/fspor.2026.1712235 (PMC12968238; doi:10.3389/fspor.2026.1712235)
Supplement: Supplementary file 1 [file Datasheet1.docx]

Appendix A Search strings

**Concepts:**

- Chronic pain
- Accelerometry
- NOT animals

**PubMed**

(("Chronic Pain"[Mesh] OR "Chronic Pain"[tiab:~5] OR "Chronic Pains"[tiab:~5]) AND ("Accelerometry"[Mesh] OR "Actigraphy"[Mesh] OR "Fitness Trackers"[Mesh] OR "Telemedicine"[Mesh] OR "Wearable Electronic Devices"[Mesh] OR Accelero*[tiab] OR Actigraph*[tiab] OR Telemetr*[tiab] OR "Fitness Tracker"[tiab:~5] OR "Fitness Trackers"[tiab:~5] OR "Fitness Tracking"[tiab:~5] OR "Activity Tracker"[tiab:~5] OR "Activity Trackers"[tiab:~5] OR "Activity Tracking"[tiab:~5] OR "Tele-Referral"[tiab] OR " Tele-Referrals"[tiab] OR "TeleReferral"[tiab] OR " TeleReferrals"[tiab] OR "Virtual Medicine"[tiab] OR "Virtual-Medicine"[tiab] OR "Mobile Health"[tiab] OR "Mobile-Health"[tiab] OR "mHealth"[tiab] OR "Telehealth"[tiab] OR "Tele-health"[tiab] OR "Wearable Electronic Device"[tiab] OR "Wearable Electronic Devices"[tiab] OR "Wearable Device"[tiab] OR "Wearable Devices"[tiab] OR "Electronic Skin"[tiab] OR "Electronic Skins"[tiab] OR "Wearable Technology"[tiab] OR "Wearable Technologies"[tiab] OR (("Movement"[Mesh:NoExp] OR "Locomotion"[Mesh] OR ((Activity[tiab] OR Activities[tiab]) AND (Physical*[tiab] OR Behaviour*[tiab] OR Behavior*[tiab] OR Motor*[tiab] OR Pattern*[tiab])) OR Movement*[tiab] OR Motion*[tiab] OR Locomotion*[tiab]) AND ("Pain Measurement"[Mesh] OR Measur*[tiab] OR Meter[tiab] OR Sensor*[tiab])))) NOT (("Adolescent"[Mesh] OR "Child"[Mesh] OR "Infant"[Mesh]) OR ("Models, Animal"[Mesh]))

**Embase**

((('Chronic pain'/exp OR (Chronic NEAR/5 Pain*):ti,ab) AND ('Accelerometry'/exp OR 'Actimetry'/exp OR 'Activity tracker'/exp OR 'Telemedicine'/exp OR 'Wearable computer'/exp OR 'Ambulatory monitoring'/exp OR (Accelero* OR Actigraph* OR Actimetr* OR Telemetr* OR ((Fitness OR Activity) NEAR/5 Track*) OR "Tele-Referral" OR "Tele-Referrals" OR "TeleReferral" OR "TeleReferrals" OR "Virtual Medicine" OR "Virtual-Medicine" OR "Mobile Health" OR "Mobile-Health" OR "mHealth" OR "Telehealth" OR "Tele-health" OR "Wearable Electronic Device*" OR "Wearable Device*" OR " Wearable computer*" OR "Electronic Skin*" OR "Wearable Technolog*" OR ((Ambulatory OR Outpatient*) NEAR/5 Monitoring)):ti,ab OR (('Movement (physiology)'/exp OR 'Motor activity'/exp OR (Movement* OR Motion* OR Locomotion* OR ((Activity OR Activities) NEAR/5 (Physical* OR Behaviour* OR Behavior* OR Motor* OR Pattern*))):ti,ab) AND ('Measurement'/exp OR 'Pain measurement'/exp OR (Measur* OR Meter OR Sensor*):ti,ab)))) NOT ([animals]/lim OR ('adolescent'/exp OR 'child'/exp))) AND [embase]/lim

**CINAHL**

((MH "Chronic Pain" OR (Chronic N5 Pain*)) AND (MH "Accelerometry+" OR MH "Actigraphy" OR MH "Fitness Trackers" OR MH "Telemedicine+" OR Accelero* OR Actigraph* OR Actimetr* OR Telemetr* OR ((Fitness OR Activity) N5 Track*) OR "Tele-Referral" OR "Tele-Referrals" OR "TeleReferral" OR "TeleReferrals" OR "Virtual Medicine" OR "Virtual-Medicine" OR "Mobile Health" OR "Mobile-Health" OR "mHealth" OR "Telehealth" OR "Tele-health" OR "Wearable Electronic Device*" OR "Wearable Device*" OR "Wearable computer*" OR "Electronic Skin*" OR "Wearable Technolog*" OR ((Ambulatory OR Outpatient*) N5 Monitoring) OR ((MH "Movement+" OR MH "Motor Activity+" OR MH "Locomotion+" OR Movement* OR Motion* OR Locomotion* OR ((Activity OR Activities) AND (Physical* OR Behaviour* OR Behavior* OR Motor* OR Pattern*))) AND (MH "Pain Measurement" OR Measur* OR Meter OR Sensor*)))) NOT ((MH "Adolescence" OR MH "Child+") OR MH "Animals")

**Psychinfo**

(Chronic N5 Pain*) AND (Accelero* OR Actigraph* OR Actimetr* OR Telemetr* OR ((Fitness OR Activity) N5 Track*) OR "Tele-Referral" OR "Tele-Referrals" OR "TeleReferral" OR "TeleReferrals" OR "Virtual Medicine" OR "Virtual-Medicine" OR "Mobile Health" OR "Mobile-Health" OR "mHealth" OR "Telehealth" OR "Tele-health" OR "Wearable Electronic Device*" OR "Wearable Device*" OR "Wearable computer*" OR "Electronic Skin*" OR "Wearable Technolog*" OR ((Ambulatory OR Outpatient*) N5 Monitoring) OR Movement* OR Motion* OR Locomotion* OR ((Activity OR Activities) N5 (Physical* OR Behaviour* OR Behavior* OR Motor* OR Pattern*)) AND (Measur* OR Meter OR Sensor*))) AND (ZZ "dissertation")

**Google Scholar**

(Chronic AND Pain*) AND (Accelero* OR Actigraph* OR Actimetr* OR Telemetr* OR ((Fitness OR Activity) AND Track*) OR "Tele-Referral" OR "Tele-Referrals" OR "TeleReferral" OR "TeleReferrals" OR "Virtual Medicine" OR "Virtual-Medicine" OR "Mobile Health" OR "Mobile-Health" OR "mHealth" OR "Telehealth" OR "Tele-health" OR "Wearable Electronic Device*" OR "Wearable Device*" OR "Wearable computer*" OR "Electronic Skin*" OR "Wearable Technolog*" OR ((Ambulatory OR Outpatient*) AND Monitoring) OR Movement* OR Motion* OR Locomotion* OR ((Activity OR Activities) AND (Physical* OR Behaviour* OR Behavior* OR Motor* OR Pattern*)) AND (Measur* OR Meter OR Sensor*)))

Appendix B Overview of results of hypotheses testing with associations


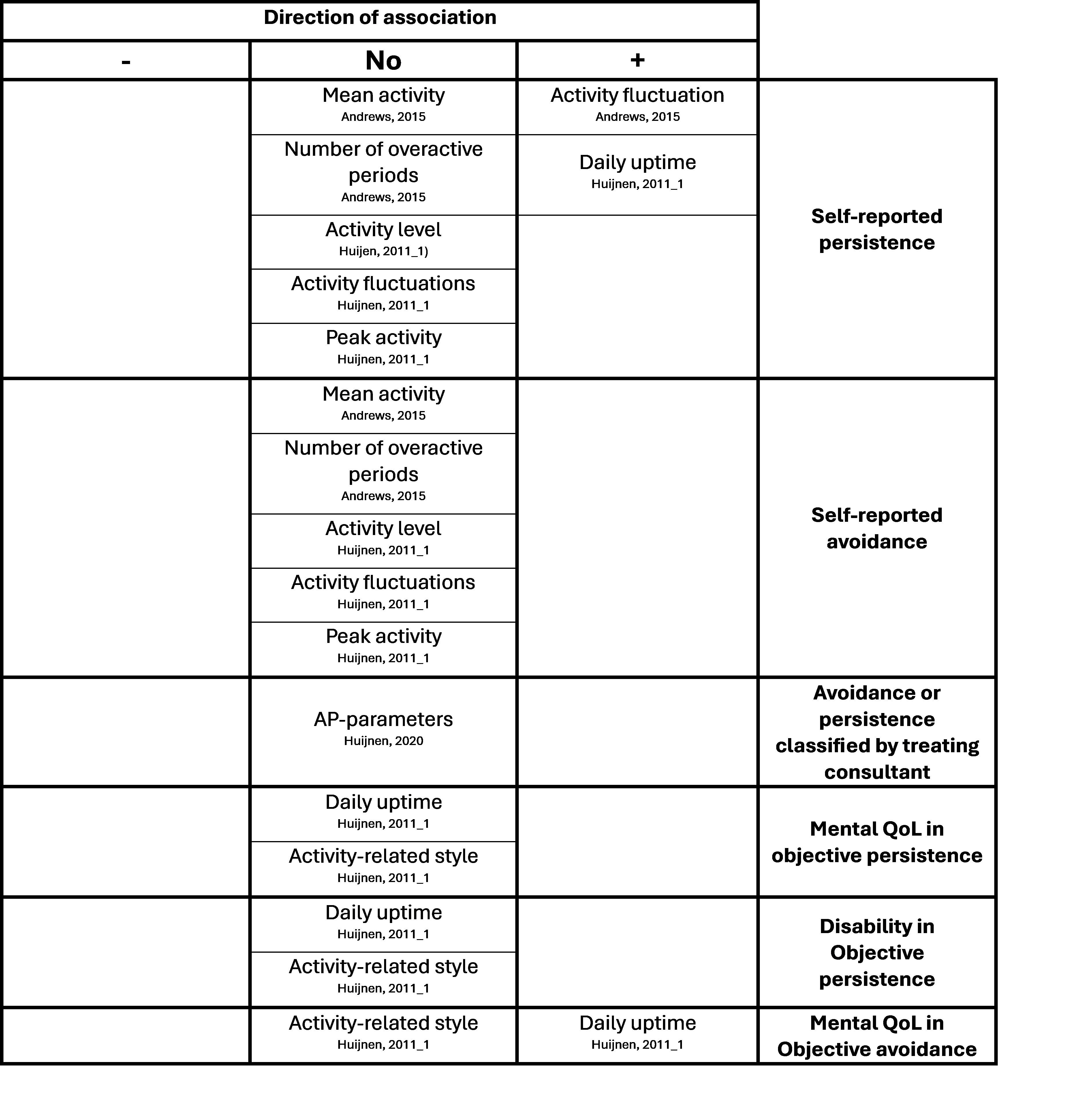


Figure B1. Significant associations (+ or -) and non-significant associations (0) between objectively measured activity pattern parameters and behavior type. + indicates a positive significant association, indicating higher scores of AP-parameters with higher scores on e.g. self-reported persistence. – indicates a negative association, indicating lower score of AP-parameters with higher score on self-reported behavior type.


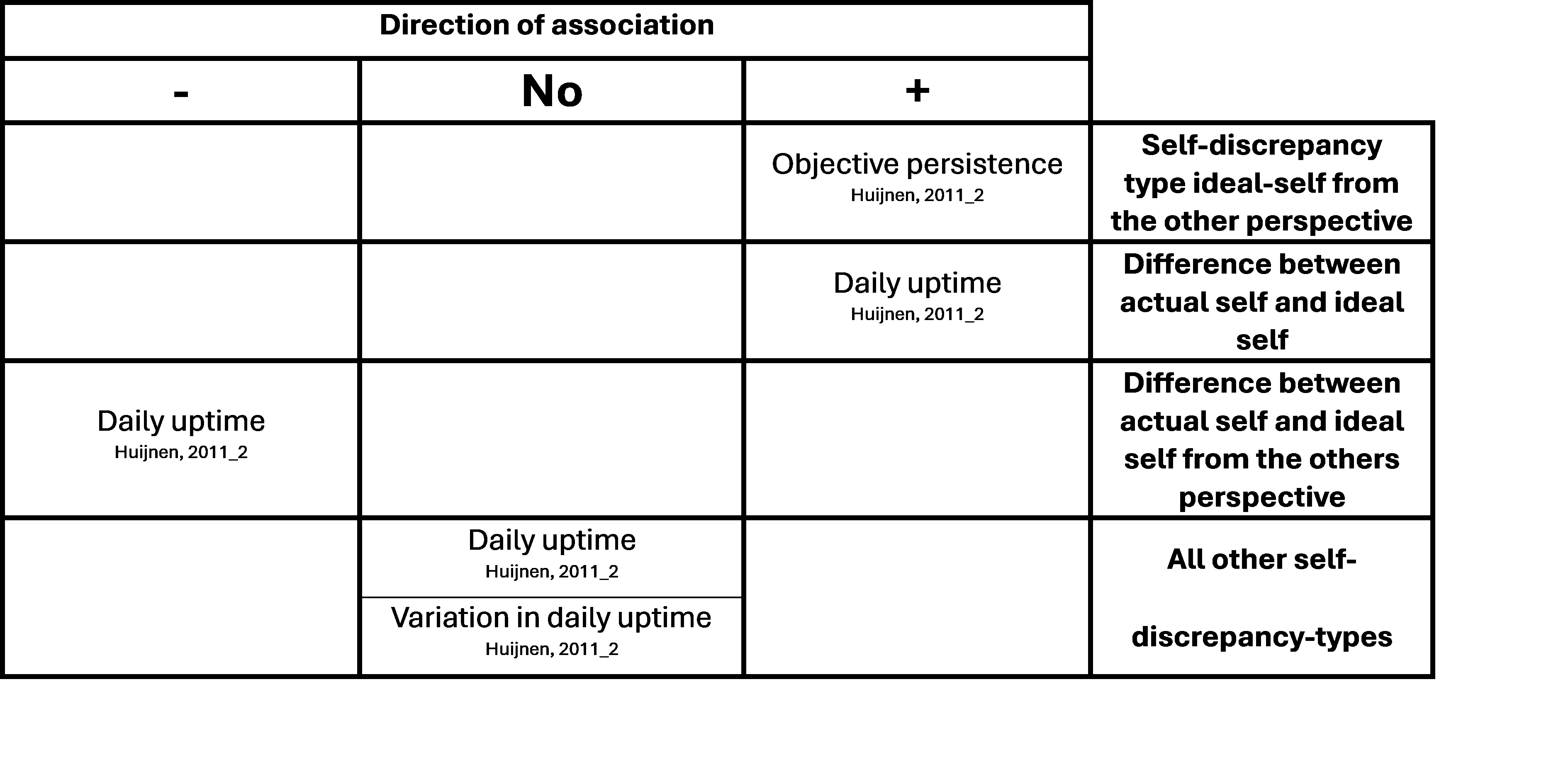
Figure B2. Significant associations (+ or -) and non-significant associations (0) between objectively measured activity pattern parameters and self-reported self-discrepancy types. + indicates a positive significant association, indicating higher scores of AP-parameters with higher scores on the self-discrepancy type e.g. self-reported persistence, – indicates a negative association, indicating lower score of AP-parameters with higher score on self-discrepancy type.


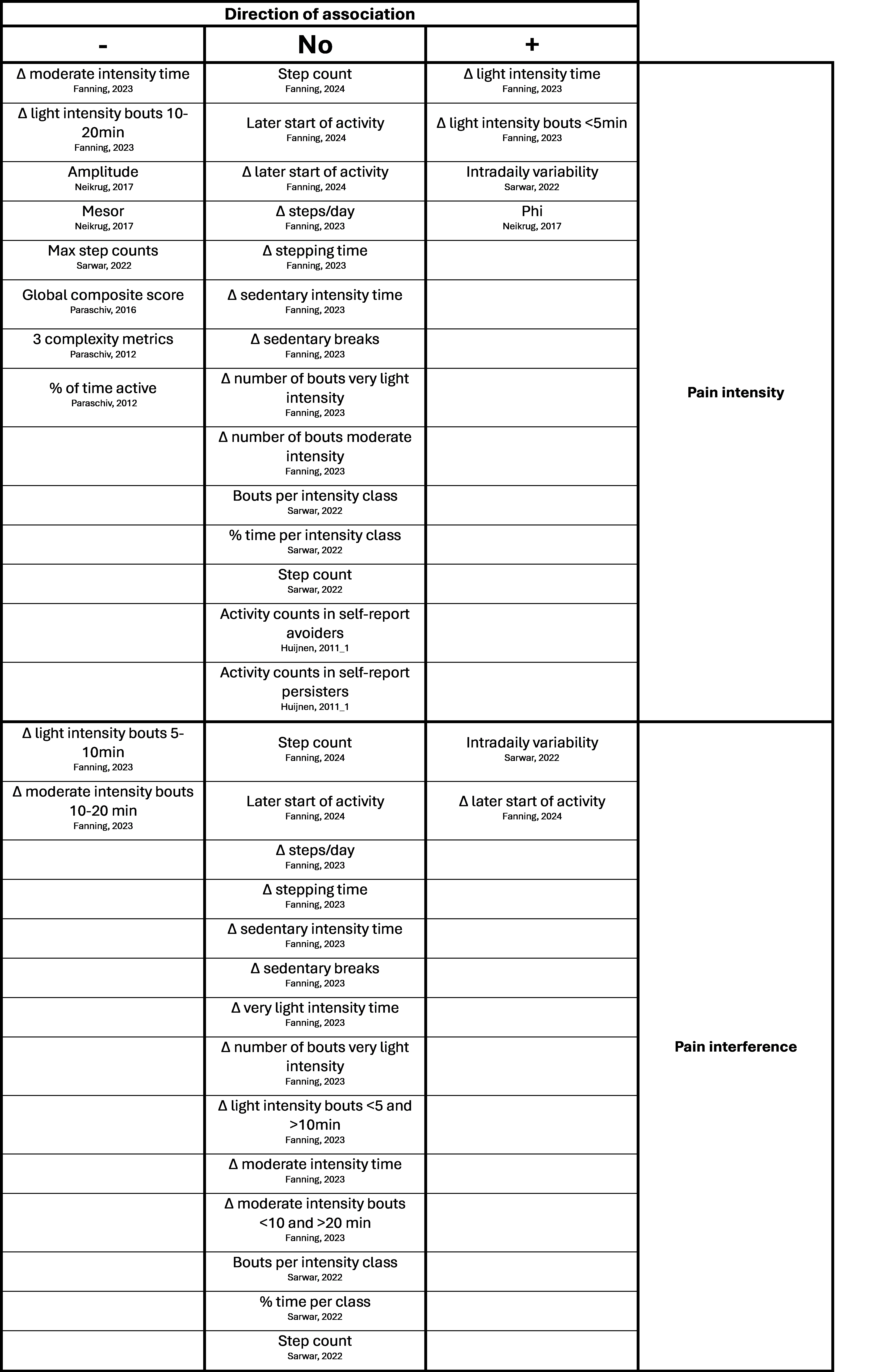


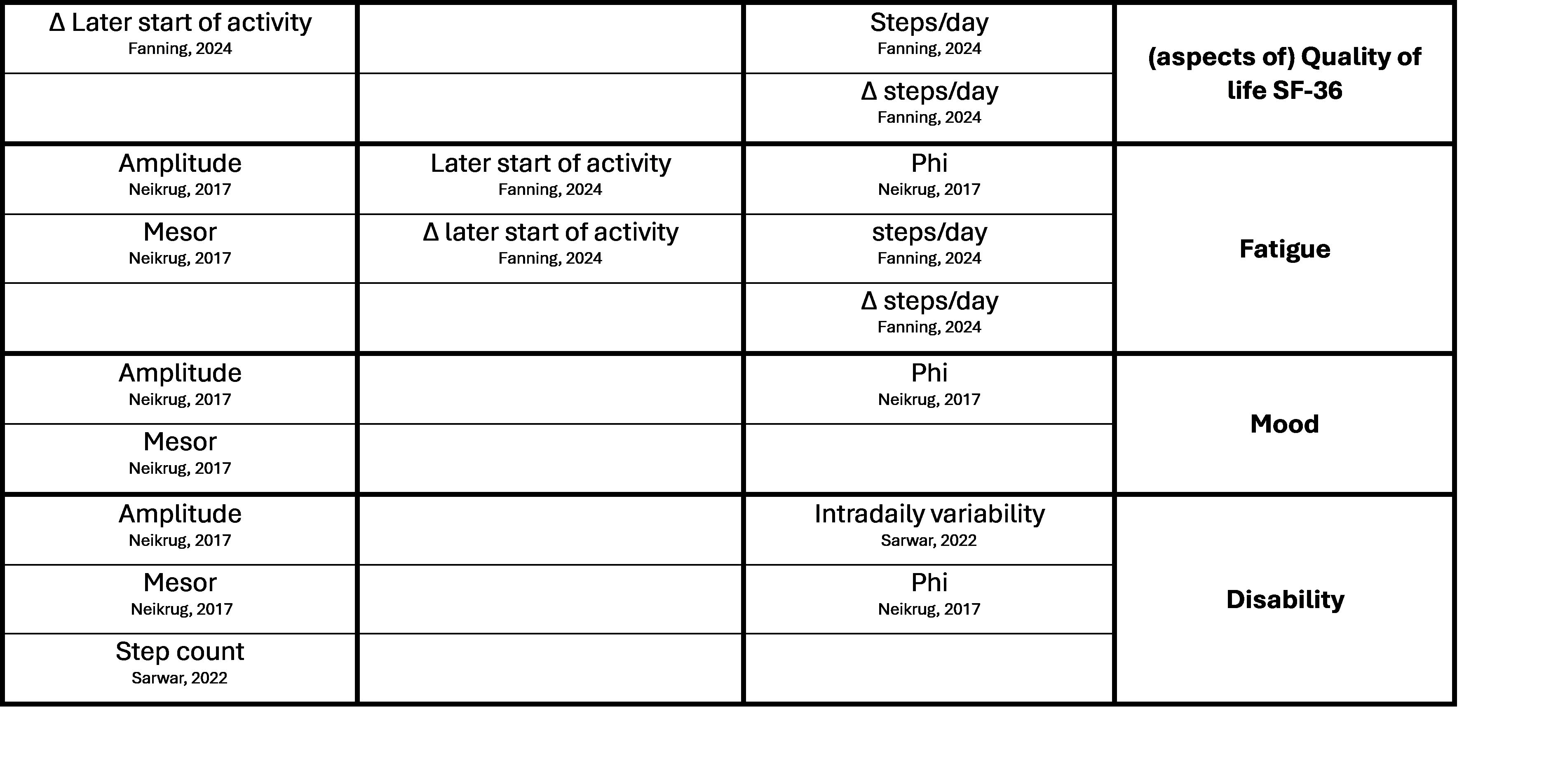
Figure B3. Significant associations (+ or -) and non-significant associations (0) between objectively measured activity pattern parameters and pain, pain interference, quality of life, fatigue, mood and disabilitiy. + indicates a positive significant association, indicating higher scores of AP-parameters with e.g. higher pain intensity, – indicates a negative association, indicating lower score of AP-parameters with e.g. higher pain intensity.
